# Two pairs of *CACNA1I* (Ca_V_3.3) variants with opposite effects on channel function cause neurodevelopmental disorders of varying severity

**DOI:** 10.1371/journal.pgen.1011828

**Published:** 2025-08-18

**Authors:** Yousra El Ghaleb, Monica L. Fernández-Quintero, Marta Campiglio, Petronel Tuluc, Ann-Sophie Höing, Fanny Kortüm, Mahdi M. Motazacker, Iris E. Jansen, Mariet W. Elting, Astrid S. Plomp, Anna-Lena M. Fischer, Victoria M. Siu, Kerstin Kutsche, Bernhard E. Flucher

**Affiliations:** 1 Institute of Physiology, Medical University Innsbruck, Innsbruck, Austria; 2 Institute of Theoretical Chemistry, University of Innsbruck, Innsbruck, Austria; 3 Department of Pharmacology and Toxicology, University of Innsbruck, Innsbruck, Austria; 4 Institute of Human Genetics, University Medical Center Hamburg-Eppendorf, Hamburg, Germany; 5 Department of Human Genetics, Amsterdam UMC, University of Amsterdam, Amsterdam, the Netherlands; 6 Department of Chemistry - BMC, Biochemistry, Uppsala University, Uppsala, Sweden; 7 Division of Medical Genetics, Department of Paediatrics, The University of Western Ontario, London, Ontario, Canada; 8 German Center for Child and Adolescent Health (DZKJ), partner site Hamburg, Hamburg, Germany; Geisel School of Medicine at Dartmouth, UNITED STATES OF AMERICA

## Abstract

The T-type voltage-gated calcium channel Ca_V_3.3 is expressed in GABAergic neurons of the thalamic reticular nucleus (TRN), where its pacemaking activity controls sleep spindle rhythmogenesis during the non-rapid eye movement (NREM) phase of natural sleep. Previously, we established *CACNA1I*, the gene coding for Ca_V_3.3, as a disease gene for neurodevelopmental disease with or without epilepsy. Here we report three newly identified activation-gate-modifying heterozygous missense variants of *CACNA1I*, found in four unrelated patients with neurodevelopmental disease with or without seizures. One of these variants, p.(Met1425Val), is an amino-acid substitution at the same position as previously published variant p.(Met1425Ile). Notably, the other two variants studied here are also a pair of two different substitutions of the same amino acid: p.(Ala398Val) and p.(Ala398Glu). By using site-directed mutagenesis, voltage-clamp electrophysiology, computational modelling of neuronal excitability, and structure modelling, we found that the two substitutions of M1425 both result in a gain of channel function including left-shifted voltage-dependence of activation and inactivation, slowed inactivation and deactivation kinetics, and increased neuronal excitability. Remarkably, the two substitutions of A398 show opposite effects on channel function. While substitution A398E leads to a gain of channel function, A398V results in decreased current density, accelerated gating kinetics, and a decreased neuronal excitability. The lack of seizures in the two independent p.(Ala398Val) patients correlates with the absence of increased neuronal excitability in this variant. This is the first report of a gate-modifying Ca_V_3.3 channel variant with partial loss-of-function effects associated with developmental delay and intellectual disability without seizures. Our study corroborates the role of Ca_V_3.3 dysfunction in the etiology of neurodevelopmental disorders. Moreover, our data suggest that substantial gain-of-function of Ca_V_3.3 leads to the development of seizures, whereas both gain- and loss-of-function variants of *CACNA1I* can cause neurodevelopmental disease.

## Author’s summary

Previously we identified *CACNA1I*, the gene coding for the T-type voltage-gated calcium channel Ca_V_3.3, as a disease gene for neurodevelopmental disease and epilepsy. That research indicated two mechanisms by which mutations of T-type calcium channels can cause such neurological diseases. Channel variants resulting in increased calcium influx, can lead to malfunction and even the death of neurons. On the other hand, variants resulting in the increased sensitivity of the channel to voltage, typically lead to neuronal hyperexcitability and seizures. Here we characterize several new disease-associated *CACNA1I* variants identified in patients with neurodevelopmental disease with and without seizures. Unexpectedly, two distinct amino acid substitutions in the same position resulted in opposite effects. Whereas A398E led to a gain of channel function, similar to the previously characterized *CACNA1I* variants, A398V resulted in decreased currents and decreased neuronal excitability. The lack of increased neuronal excitability of this variant correlates with the absence of seizures in the two independent p.(Ala398Val) patients. Thus, our findings highlight the idea that both excessive and insufficient Ca_V_3.3 channel activity can have harmful effects. While substantial gain-of-function of Ca_V_3.3 leads to the development of seizures, both gain- and loss-of-function variants of *CACNA1I* can cause neurodevelopmental disease.

## Introduction

Low-voltage-gated (LVA) calcium channels (Ca_V_3.1-Ca_V_3.3), also known as T-type channels, are central regulators of neuronal activity and promising drug targets for the treatment of pain and epilepsy [1]. These channels play a crucial role in modulating repetitive firing and pacemaker currents in neuronal networks. Specifically, Ca_V_3.3 is expressed in GABAergic neurons of the thalamic reticular nucleus (TRN), where it contributes to the regulation of rhythmic activity in the thalamocortical network. In particular, the pacemaking activity of Ca_V_3.3 is essential for the generation of sleep spindle rhythmogenesis, during the non-rapid eye movement (NREM) phase of natural sleep [2,3].

Earlier, the Ca_V_3.3-encoding gene *CACNA1I* was described as a risk gene for schizophrenia and migraine [4–7, reviewed in 8]. More recently, based on four distinct gain-of-function variants in the channel activation gate, we identified *CACNA1I* as a disease gene for autosomal dominant neurodevelopmental disorders [9]. The heterozygous p.(Ile860Met) variant, identiﬁed in three family members with variable cognitive impairment, and the *de novo* p.(Ile860Asn) variant, detected in a patient with severe developmental delay and seizures, changed an amino acid at the cytoplasmic end of the IIS6 helix. In addition, the *de novo* variants p.(Ile1306Thr) and p.(Met1425Ile), found in individuals with severe developmental delay, early-onset epilepsy, severe speech impairment, and cortical visual impairment, affected residues at the cytoplasmic ends of IIIS5 and IIIS6, respectively. Electrophysiological analysis of Ca_V_3.3 channels expressed in HEK293T cells revealed that all amino acid substitutions slowed the kinetics of current activation, inactivation and deactivation as well as shifted the voltage dependence of activation and inactivation to more negative membrane potentials. These changes of the gating properties resulted in increased calcium influx at rest and during repetitive firing, potentially leading to calcium toxicity. Computational modelling of TRN neurons demonstrated that the altered gating properties lowered the threshold and increased the frequency and duration of neuronal firing, which could explain the observed seizure phenotype. Both of these effects represent a gain of channel function. Importantly, the magnitude of the changes in channel gating properties correlated with the TRN neuron model hyperexcitability as well as the severity of the disease phenotype. Interestingly, substitution of the same residues with two different amino acids (I860N and I860M) resulted in distinct gating alterations, underlying neurological phenotypes of varying severity in different patients [9].

**Fig 1:**
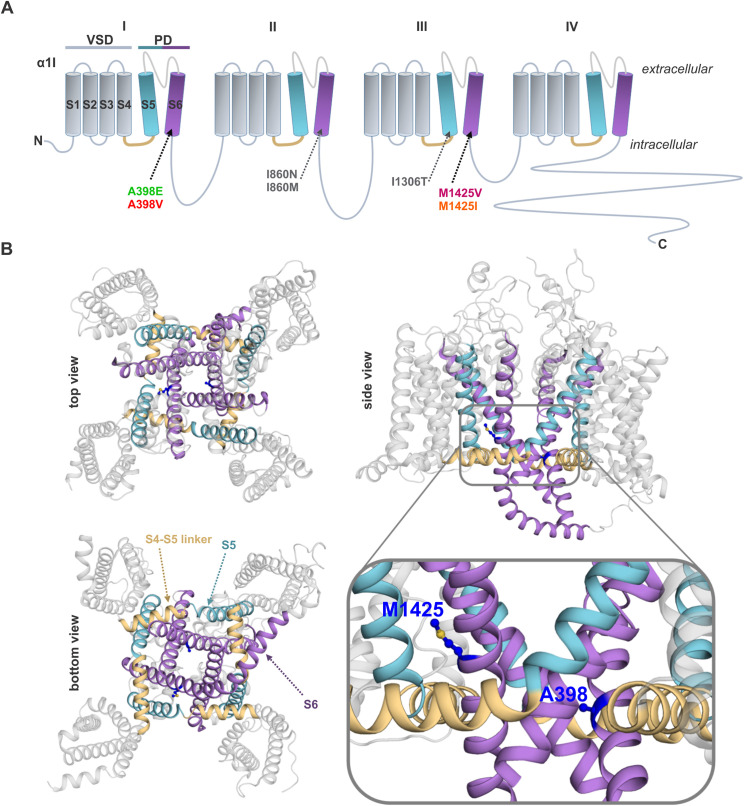
Location of disease-associated *CACNA1I* amino acid substitutions in the domain structure and structure modelling of Ca_V_3.3. Voltage sensor domain (VSD) helices S1-S4 displayed in grey, connecting S4-S5 linker in beige, and pore domain (PD) helices S5 in cyan and S6 in purple. **(A)** Domain structure of Ca_V_3.3 indicating the location of the disease-associated variants at the cytoplasmic end of S6 helices. A398E (green) and A398V (red) in IS6, M1425V (magenta) and M1425I (orange) in IIIS6. Indicated in grey are also the three previously published *CACNA1I* disease-associated amino acid substitutions I860N and I860M in IIS6, and I1306T in IIIS5 (El Ghaleb et al., 2021) [9]. **(B)** Top-, bottom-, and side view of the structure model of Ca_V_3.3 α1 subunit based on the cryo-EM structure of Ca_V_3.3 (PDB accession code: 7WLI) in the inactivated state with a closed channel gate. The inset shows a zoom-in with the two changed residues A398 and M1425 displayed.

Here we report three newly identified activation-gate-modifying disease variants of *CACNA1I*, found in four unrelated patients with neurodevelopmental disorders, with or without seizures. These three variants each affect an amino acid residue located at the cytoplasmic end of the S6 helix (Fig 1A and 1B). This region is known to be a hotspot for disease-associated variants in the calcium channel family [10]. The cytoplasmic ends of the four homologous S6 helices together with the S5 and S4-S5 linkers form the activation gate of the channel that opens upon depolarization and allows calcium ion influx [11]. Variants A398E and A398V affect the same amino acid residue in the S6 helix of the first domain of Ca_V_3.3. The third newly identified variant, M1425V, affects an amino acid residue in the S6 helix of domain three. Remarkably, a substitution of the same amino acid residue was found in one of our previously described pathogenic variants [9]. Therefore, the newly identified *CACNA1I* disease-associated variants provide us with the opportunity to study two pairs of different amino acid substitutions at the same positions.

Using a combination of structural modelling, site-directed mutagenesis, electrophysiology, and computational modelling of neuronal excitability, we show that variants M1425V and A398E display characteristic gain of channel function effects, similar to what we described previously [9]. The variants resulted in hyperpolarizing shifts in the voltage dependence of activation and inactivation and slowed down inactivation and deactivation kinetics. The resulting alterations led to increased calcium influx at resting membrane potentials, which in turn heightened neuronal excitability, explaining the epilepsy and impaired neurodevelopmental phenotype. Interestingly, variants A398V and A398E show opposite effects on channel conformation and gating. This is the first report of a Ca_V_3.3 channel disease-associated variant (A398V) demonstrating a partial loss of channel function linked to developmental delay and intellectual disability without seizures. Our study corroborates the role of Ca_V_3.3 dysfunction in the etiology of neurodevelopmental disorders with and without epilepsy. In addition, our data suggest that substantial gain-of-function of Ca_V_3.3 leads to the development of seizures, whereas both gain- and loss-of-function variants of *CACNA1I* can cause neurodevelopmental disorder.

## Results

### Four unrelated patients with a neurodevelopmental disorder and a heterozygous *CACNA1I* missense variant

We identified four unrelated patients with three different novel heterozygous missense variants in *CACNA1I* (mRNA reference sequence NM_021096.4). The genetic and clinical findings of the four patients in are summarized Table 1. Trio exome sequencing in patients 1, 2, and 3 and their healthy parents revealed the *de novo* heterozygous c.1193C > A; p.(Ala398Glu) variant in patient 1 and the same *de novo* heterozygous c.1193C > T; p.(Ala398Val) variant in patients 2 and 3. In patient 4, targeted gene panel sequencing identified the heterozygous c.4273A > G; p.(Met1425Val) variant that was absent in the mother. The variant c.4273A > G; p.(Met1425Val) is absent in the gnomAD population database (v4.1.0), while the variant c.1193C > T; p.(Ala398Val) is present in 4 out of 1,570,412 alleles (minor allele frequency of 0.0003%). The third variant c.1193C > A; p.(Ala398Glu) was identified in a single individual, however, the allele balance for the adenine was in the range of 0.2-0.25 suggesting somatic mosaicism of the variant in the analyzed tissue/cells [12]. The pathogenicity prediction programs AlphaMissense, CADD, and REVEL predicted the three missense variants to have a deleterious impact on protein function with high scores [13–15].

**Table 1:** Clinical characteristics of patients with *CACNA1I* missense variants.

	Patient #	1	2	3	4
**Variant**	NM_021096.4 NP_066919.2	c.1193C > A p.(Ala398Glu)	c.1193C > T p.(Ala398Val)	c.1193C > T p.(Ala398Val)	c.4273A > G p.(Met1425Val)
	Exon	8	8	8	25
	Origin	*de novo*	*de novo*	*de novo*	Not present in mother, father not available for sequencing; father has grade 9 education, significant history of learning difficulties; attention difficulties in fathers extended family
**Evaluation**	Nationality	Asian	Caucasian	Pakistani	Caucasian
	Sex	Male	Female	Male	Male
**Pregnancy and birth**	Pregnancy	Uneventful	Uneventful	Uneventful	Uneventful
	Birth at (weeks)	40	41	40	38
	Birth weight (z-score)	2900 g (-1.5 z)	2840 g (-1.72 z)	Unknown	3175 g (-0.31 z)
	Birth length (z-score)	ND	51 cm (-0.55 z)	Unknown	Unknown
	OFC birth (z-score)	ND	32 cm (-2.46 z)	Unknown	Unknown
**Last examination**	Age	2 y 3 m	13 y	4 y	25 y
	Weight (z-score)	ND (normal build)	39.5 kg (-1.15 z)	18 kg (0.51 z)	81.6 kg (+1.0 z) (at age 7.5 y: 26.5 kg; + 0.47 z)
	Height (z-score)	91.5 cm (+0.12 z)	154 cm (-0.76 z)	108.5 cm (+1.01 z)	178 cm (0 z) (at age 7.5 y: 124 cm; -0.34 z)
	OFC (z-score)	48.5 cm (-0.71 z)	50 cm (-3.6 z)	48 cm (-2.51 z)	52.8 cm (-2.8 z)
**Development**	Developmental delay/intellectual disability	Severe global developmental delay	Intellectual disability	Developmental delay, toilet training not successful	Early developmental milestone appropriate; moderate intellectual disability
	Motor development	Delayed, sitting alone at 14 m, rolling over at 18 m, pull up to stand at 2 y	Normal (is not able to walk long distances because of tip-toe gait and consecutive pain)	Normal (walking at 15 m)	Sat at 4 m, walked at 10 m, concern about balance and coordination at 2 y
	Speech impairment	No speech	Receptive and expressive language delay, deficit in grammar	No speech	Single words at 10 m; at 6 y, expressive and receptive language skills in normal range
**Neurological features**	Abnormal muscle tone	Truncal hypotonia	No	No	Rigidity
	Seizures	Yes	No	No	None documented but has had staring spells for most of his life, up to 9 seconds in duration
	Seizure onset	23 m	NA	NA	NA
	Seizure type	Generalised tonic clonic	NA	NA	NA
	EEG	Electrical status epilepticus during slow-wave sleep at 4 y	Normal at 13 y; normal in long-term EEG at 15 y	NA	Normal at age 13 y and 27 y
	Response to treatment	Yes, clobazam	NA	NA	Has been on multiple medication’s to control his behaviour abnormalities
	MRI scan	Normal at 18 m	Normal at 13 y	NA	Right temporal lobe cyst, no mass effect at 23 y
	Sleep abnormality	No	No	No	Decreased need for sleep as infant; since 10 y of age: insomnia
	Abnormality of mental function	Autistic and stereotypic behaviour	No	Possible diagnosis: attention deficit hyperactivity disorder, autistic behaviour	Attention deficit hyperactivity disorder; staring spells (for most of his life); since 15 y of age: episodes of aggressive behavior, irritability, agitation; since 17 y of age: visual and vivid hallucinations, psychosis, posttraumatic stress symptom
**Other features**	Hearing	Normal	No recent hearing testing performed, subjectively no hearing impairment	Normal (tympanogram and otoacoustic emissions)	Hyperacusis
	Vision	Normal	Myopia	Normal	Exotropia and mild ptosis, excellent vision
	Feeding	Impaired mastication	Normal	Mild problems, no interest in fruit or vegetables	Low gag threshold, spits up easily
	Abnormality of the face	Plagiocephaly, triangular face, anteverted ears, synophrys, tented upper lip vermilion	Arched eyebrows, protruding ears, prominent nose	Mild brachycephaly, triangular face, synophrys, protruding ears	High nasal bridge, prominent nasal tip
	Other findings	3 café-au-lait spots, hyperpigmentation of neck and axillae	Reduced social reciprocity, wore diapers until the age of 12 y	3 small café-au-lait spots	Broad thumbs, long toes, small hepatic hemangioma

Abbreviations: EEG, electroencephalogram; m, months; MRI, magnetic resonance imaging; NA, not applicable; ND, no data; OFC, occipital frontal circumference; y, years; z, z-score.

The 2-year-old male patient 1 with the p.(Ala398Glu) variant had severe global developmental delay, absent speech, hypotonia, and autistic and stereotypic behaviour. At the age of 23 months, he developed generalised tonic clonic seizures that were controlled with medication (see Table 1). Female patient 2 (13 years old) and male patient 3 (4 years old) with the p.(Ala398Val) variant had a milder phenotype than patient 1. Both had microcephaly, developmental delay (DD) or intellectual disability (ID), delayed language development or absent speech, but normal motor development and no seizures. The 25-year-old male patient 4 with the p.(Met1425Val) variant had microcephaly, moderate ID, attention deficit hyperactivity disorder, rigidity, and hyperacusis. He did not have seizures but staring spells throughout his life. He showed sleep abnormalities and mental function changes. Since the age of 10 years, he had insomnia. Aggressive behaviour, irritability, and agitation started at the age of 15 years. Since the age of 17 years, he had hallucinations and psychosis (Table 1).

### The *CACNA1I* variants have gain- and loss-of-function effects on the current amplitude and voltage dependence of activation

**Fig 2:**
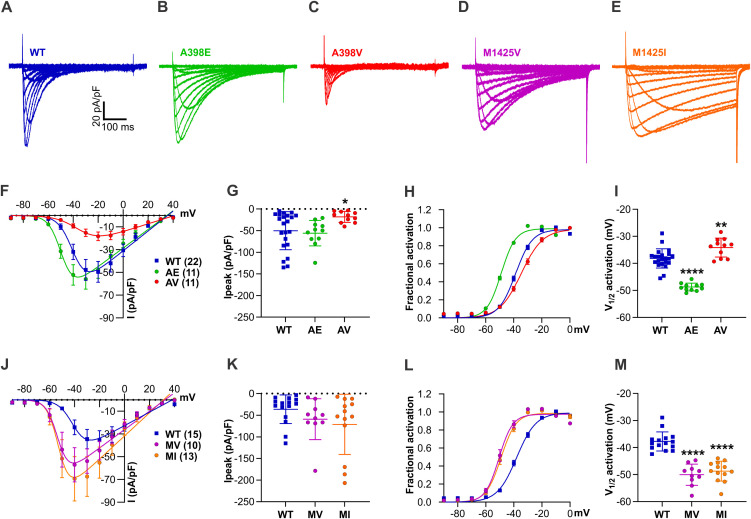
Distinct effects on calcium channel activation of *CACNA1I* disease-associated amino acid substitutions A398E/V and M1425V/I. **(A-E)** Representative calcium current recordings in response to 500 ms depolarization to voltages between -90 mV to +40 mV in 10 mV increments for **(A)** Ca_V_3.3 wild-type (WT) in blue, **(B)** A398E (AE) in green, **(C)** A398V (AV) in red, **(D)** M1425V (MV) in magenta, (**E**) and M1425I (MI) in orange. **(F, J)** The current-voltage relationship of the two A398 variants (**F**) and the two M1425 variants **(J)**, each set compared to matched WT controls. **(G, K)** Peak current densities are not altered in AE (p = 0.8791), MV (p = 0.4624), and MI (p = 0.1475), except in AV, where they are reduced by more than 2-fold (p = 0.0348). **(H, L)** Fractional activation curves and **(I, M)** V_1/2_ of activation scatterplots show significantly left-shifted voltage-dependence of activation for Ca_V_3.3 AE by 10.7 mV, MV by 12.3 mV, and MI by 11.0 mV. The V_1/2_ of AV is right-shifted by 4.0 mV, all as compared to the respective wild-type controls. Mean ± SEM; p-values calculated with one-way ANOVA and Dunnett’s multiple comparisons test; * p < 0.05, ** p < 0.01, **** p < 0.0001.

We expressed the three newly identified *CACNA1I* amino acid substitutions (A398E, A398V, M1425V), alongside our previously published M1425I variant [9] and wild-type controls in HEK293T cells and performed whole-cell patch clamp recordings in order to study the effects of the amino acid substitutions on the biophysical gating properties of Ca_V_3.3. We conducted two sets of experiments: In the first set, we compared the current properties of the two substitutions of A398 (A398E, A398V) with wild-type controls and in the second set we compared the two substitutions of M1425 (M1425V, M1425I) with separate wild-type controls (Fig 2). For comparison across datasets, see supplementary figure and table with pooled wild-type controls (S1A–D Fig and S1 Table). Representative example current traces of each Ca_V_3.3 construct are shown in Fig 2A–E. All variants express calcium currents, albeit with significantly differing amplitudes and gating properties. The current/voltage (I/V) curve and fractional activation plotted against the voltage of the depolarizing test pulse show that the glutamate substitution of A398 (A398E) resulted in a significant 10.7 mV left shift of the voltage dependence of activation (V_½_) to more hyperpolarizing voltages with no change in current density. On the contrary, the valine substitution (A398V) of the same residue showed an almost 3-fold reduction in current density and a 4.0 mV right-shifted V_1/2_ of activation (voltage at which half of the channels is activated). The latter resulted mainly from the increased slope factor (k) of the A398V I/V curve (Fig 2F–I and Table 2). For the two substitutions of M1425, the direction and severity of the effects were comparable to each other, as well as with those of A398E. Substituting M1425 with valine (M1425V) or isoleucine (M1425I) both resulted in a significant hyperpolarizing shift of the activation curve, by 12.3 mV and 11.0 mV, respectively. No significant differences in current density were found with either one of the M1425 substitutions (Fig 2J–M and Table 2).

**Table 2:** Activation gating properties of *CACNA1I* variants.

	Ca_V_3.3-WT	p-value one-way ANOVA	Ca_V_3.3-A398E	Dunnett’s adjusted p-value	Ca_V_3.3-A398V	Dunnett’s adjusted p-value
**Ipeak (pA/pF)**	-51.1 ± 9.3	0.0276*	-55.8 ± 7.6	0.8791	-18.2 ± 3.3	0.0348
**Gmax (nS/nF)**	856.4 ± 164	0.0861	739.5 ± 106.6	0.8325	351.1 ± 65.9	0.0545
**V1/2 act. (mV)**	-38.1 ± 0.8	<0.0001****	-48.8 ± 0.4	<0.0001	-34.1 ± 0.9	0.0026**
**k act. (mV)**	6.1 ± 0.3	<0.0001****	5.2 ± 0.1	0.1510	8.0 ± 0.5	0.0004***
**Vrev (mV)**	43.5 ± 3.3	0.9372	44.8 ± 1.5	0.9441	43.0 ± 1.7	0.9931
**n**	22	--	11	--	11	--
	**Ca**_**V**_ **3.3-WT**	**p-value one-way ANOVA**	**Ca**_**V**_ **3.3-M1425V**	**Dunnett’s adjusted p-value**	**Ca**_**V**_ **3.3-M1425I**	**Dunnett’s adjusted p-value**
**Ipeak (pA/pF)**	-36.2 ± 10.1	0.2041	-59.2 ± 12.2	0.4624	-71.3 ± 29.9	0.1475
**Gmax (nS/nF)**	708.6 ± 226.7	0.3301	871.4 ± 166.7	0.8688	1211.2 ± 504.1	0.2493
**V1/2 act. (mV)**	-37.7 ± 1.2	<0.0001****	-50.0 ± 1.0	<0.0001****	-48.7 ± 1.4	<0.0001****
**k act. (mV)**	7.0 ± 0.5	0.0003***	4.7 ± 0.2	0.0005***	5.1 ± 0.4	0.0024**
**Vrev (mV)**	36.0 ± 2.7	0.0470*	30.9 ± 1.5	0.2343	28.2 ± 2.5	0.0298*
**n**	15	--	10	--	13	--

Displayed are means±SEM, Dunnett’s adjusted p-values of one respective Ca_V_3.3 variant vs. WT, and the one-way ANOVA p-values of WT vs. the two respective Ca_V_3.3 variants (WT vs. AE/AV or WT vs. MV/MI). *p < 0.05, **p < 0.01, ****p < 0.0001

In summary, the left-shifted voltage dependence of activation of A398E, M1425V, and M1425I allows these channels to activate closer to the resting membrane potential of the neurons in which they are expressed; thus indicating a gain-of-function effect. For A398V, the slight but significant right shift together with the almost 3-fold current reduction compared to wild-type Ca_V_3.3 currents suggests a partial loss-of-function phenotype potentially resulting in a severe reduction of calcium influx.

### The *CACNA1I* variants differentially effect inactivation and deactivation kinetics

**Fig 3:**
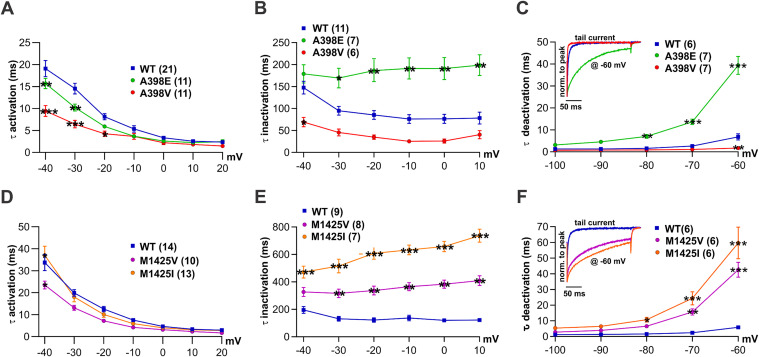
Activation, inactivation, and deactivation kinetics differ between Ca_V_3.3 WT and variants as well as among the variants. **(A, D)** Time constants of activation calculated from fits of the rising phase during 500 ms step depolarisations to the indicated voltages. **(A)** Both A398E (green) and A398V (red) activate faster between -40 and -20 mV, as compared to the WT (blue). **(D)** At -30 mV and higher depolarisations both substitutions of M1425 activate as fast as WT. **(B and E)** Time constants of inactivation determined by fitting the decay phase of currents during 5 s depolarisations. **(B)** AE inactivates slower and AV faster, compared to WT. **(E)** Both M1425V (magenta) and M1425I (orange) inactivate slower compared to WT. **(C and F)** Time constants of deactivation determined from tail current decay at indicated repolarising voltages after 15 ms pulse to V_max_. Insets display representative normalized example traces. **(C)** A398E deactivates increasingly slower, A398V faster only at -60 mV. **(F)** M1425V and M1425I deactivate increasingly faster compared to WT. Mean ± SEM; p-values calculated with repeated measures ANOVA and the Holm-Sidak test for multiple comparisons. *p < 0.05, **p < 0.01, ****p < 0.0001.

To determine the activation kinetics of the four different Ca_V_3.3 variant channels, we analyzed the rising phase of currents elicited by 500 ms depolarizing pulses at incrementally increasing voltages from a holding potential of -100 mV. The data were fitted using a mono-exponential function. Since the kinetics of T-type calcium channels are strongly voltage-dependent, we compared the average time constant of activation of the variants with that of their respective WT controls at each voltage (Fig 3A and 3D). For comparison across datasets, see supplementary figure with pooled wild-type controls (S1E–G Fig). The representative example recordings (Fig 2A–C) and the quantitative analyses (Fig 3A) show that both variants of A398 activated with a speed similar to that of wild-type Ca_V_3.3 at depolarizations higher than -20 mV, and for the two substitutions of M1425 activation kinetics were similar to wild-type from depolarization of -30 mV and higher (Figs 3D, 2D and 2E).

The inactivation kinetics showed robust differences between wild-type Ca_V_3.3 and the variants at all voltages. Since 500 ms test pulses were not sufficiently long to achieve the maximum inactivation of several of the examined Ca_V_3.3 variants, inactivation kinetics were analyzed by fitting the decaying phase of 5 s current recordings using a mono-exponential function. At all voltages, the two substitutions of A398 show opposite effects (Figs 3B and 2A-C). A398V showed a loss-of-function effect with an approximately 2-fold faster inactivation compared to WT controls, leading to faster channel closing and reduced total calcium influx. However, this effect was only statistically significant at -40 mV (Fig 3B). In contrast, A398E showed a gain-of-function effect as it inactivated up to 2-fold slower compared to WT, resulting in prolonged channel openings and increased calcium influx. Similar gain-of-function effects were observed for the two substitutions of M1425 (Figs 3E, 2D and 2E). M1425V inactivated up to 2-fold and M1425I 4-fold slower compared to wild-type Ca_V_3.3 channels.

The same pattern was detected when studying the deactivation kinetics of the Ca_V_3.3 variants. In order to analyze the speed of channel closing upon repolarization, cells were held at -100 mV and depolarized for 15 ms to the voltage of maximal current activation (V_max_). This pulse is short to minimize the effects of inactivation on the tail currents. The time constant of deactivation was determined with a mono-exponential fit on the decaying phase of the tail current at repolarizing voltages between -100 mV and -60 mV. A gain-of-function effect of slowed deactivation was observed for A398E, M1425I and M1425V (Fig 3C and 3F). Deactivation of wild-type Ca_V_3.3 became slower at repolarizations to less negative potentials. This phenomenon was exaggerated for the variants A398E, M1425V, and M1425I. Interestingly, the deactivation kinetics of A398V continued to be fast even at -70 and -60 mV, where Ca_V_3.3-WT deactivation started to slow down. This resulted in significantly faster deactivation for A398V at -60 mV, as compared to its WT controls (Fig 3C).

### All *CACNA1I* variants left-shift steady state inactivation and shift or abolish the window current

**Fig 4:**
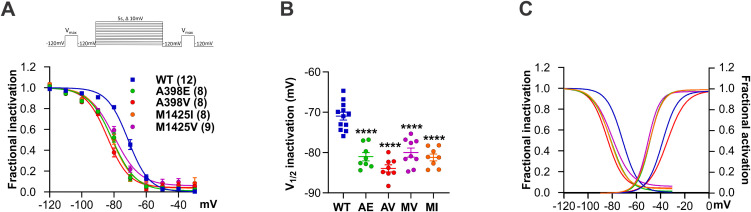
All Ca_V_3.3 variants left-shift the voltage dependence of inactivation. Inset in **A** shows the steady-state inactivation protocol used for these experiments. **(A)** The fractional inactivation curves (**B**) and scatterplot of the V_1/2_ of the voltage dependence of inactivation show left shifts for each of the four variants: 10.1 mV for A398E (AE, green), 13.0 mV for A398V (AV, red), 9.1 mV for M1425V (MV, magenta), and 10.3 mV for M1425I (MI, orange). **(C)** Superimposing fractional inactivation and activation curves (WT activation curve from pooled WT data S1C Fig) demonstrates left-shifted activation and inactivation together result in left-shifted window-currents for AE (green), MV (magenta), and MI (orange), compared to the respective WT control (blue). For AV (red), only the inactivation curve is left-shifted and the activation curve right-shifted, resulting in a reduced window-current voltage-range. Mean ± SEM; p-values calculated with one-way ANOVA and Dunnett’s multiple comparisons test; **** p < 0.0001.

To examine whether the Ca_V_3.3 variants also cause altered channel availability at different voltages, we measured the steady-state inactivation. Therein, we compared the current amplitude in response to a pre-pulse at V_max_, with the current amplitude to a test pulse at V_max_ after a 5 s conditioning pulse with incrementally increasing voltage steps from -120 mV to -30 mV (see inset in Fig 4A). As opposed to the effects on the kinetics of inactivation and deactivation, and on the voltage dependence of activation, the direction of the effect on voltage dependence of inactivation was the same in all tested variants. We observed a 10.1 mV left-shifted inactivation curve in A398E and an even stronger left-shift of 13.0 mV in A398V, as compared to Ca_V_3.3 WT (Fig 4A and 4B and Table 3). The two substitutions of M1425 both left-shifted the voltage dependence of inactivation with 9.1 mV shift for M1425V and a 10.3 mV shift for M1425I (Fig 4A and 4B and Table 3).

**Table 3:** Inactivation gating properties of *CACNA1I* variants.

	Ca_V_3.3-WT	p-value one-way ANOVA	Ca_V_3.3-A398E	Dunnett’s adjusted p-value	Ca_V_3.3-A398V	Dunnett’s adjusted p-value	Ca_V_3.3-M1425V	Dunnett’s adjusted p-value	Ca_V_3.3-M1425I	Dunnett’s adjusted p-value
**V1/2 inact. (mV)**	-70.9 ± 1.0	<0.0001 ****	-81.0 ± 1.0	<0.0001 ****	-83.9 ± 0.9	<0.0001 ****	-80.0 ± 1.1	<0.0001 ****	-81.2 ± 0.9	<0.0001 ****
**k inact. (mV)**	6.2 ± 0.3	0.0029**	7.2 ± 0.4	0.0024**	6.6 ± 0.4	0.0339*	7.7 ± 0.7	0.0005***	7.0 ± 0.5	0.0025**
**n**	12	--	8	--	8	--	9	--	8	--

Displayed are means±SEM, Dunnett’s adjusted p-values of one respective Ca_V_3.3 variant vs. WT, and the one-way ANOVA p-values of WT vs. the four Ca_V_3.3 variants. *p < 0.05, **p < 0.01, ***p < 0.001, ****p < 0.0001

Superimposing the inactivation and activation curves allows us to estimate the voltage-range and magnitude of the persistent window current (Fig 4C). For the A398E, M1425V, and M1425I variants, the left-shifted inactivation curves were paralleled by similar left-shifts of the activation curves, thus resulting in a left-shifted window-current of comparable magnitudes compared to wild-type Ca_V_3.3. In contrast, in A398V the left-shifted inactivation was not accompanied by a similar shift in the activation, thus narrowing the voltage-range of the window-current. This further supports the notion that the A398V variant causes a Ca_V_3.3 loss-of-function phenotype with reduced calcium influx at rest.

**Fig 5:**
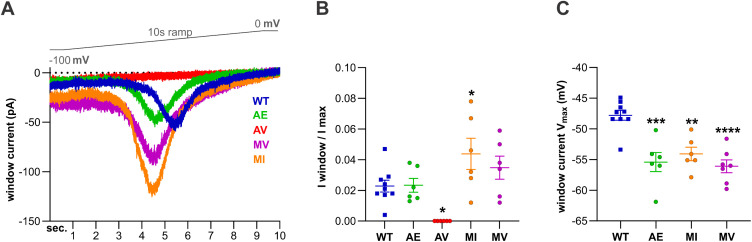
Three Ca_V_3.3 variants left-shift the window current, while the A398V completely abolishes the window current. **(A)** Representative example traces of a 10 sec. ramp recording from -100 mV to 0 mV of Ca_V_3.3 wild-type (WT, blue), A398E (AE, green), A398V (AV, red), M1425V (MV, magenta), and M1425I (MI, orange). Note that the ramp voltage (gray) increases parallel to the time. **(B)** While the size of the window current, normalized against the peak current density (I window/ I max) is unchanged for AE and not significantly increased in the MV variant, the MI variant shows a significant 2-fold increase of the relative window current. The window current of the AV variant is completely absent. **(C)** As compared to WT controls, the three variants AE (7.6 mV shift), MV (8.3 mV shift), and MI (6.3 mV shift) all show significantly left-shifted peaks of their window current. Mean ± SEM; p-values calculated with one-way ANOVA and Dunnett’s multiple comparisons test; *p < 0.05, **p < 0.01, ***p < 0.001, ****p < 0.0001.

To directly measure the window-current, we performed a 10 s ramp protocol from -100 mV to 0 mV (Fig 5). As expected A398E, M1425V, and M1425I all showed a significantly left-shifted peak of their window current (Fig 5A and 5C and Table 4). In addition, for M1425I the window-current peak was significantly increased by 2-fold compared to Ca_V_3.3 WT (Fig 5B and Table 4). M1425V also showed an increased window current however, the difference remained below statistical significance. In contrast, for A398V the window-current was completely abolished (Fig 5A and 5B). This was due to its substantially reduced current density, accelerated inactivation, and left-shifted voltage dependence of inactivation.

**Table 4:** Window current properties.

	Ca_V_3.3-WT	p-value one-way ANOVA	Ca_V_3.3-A398E	Dunnett’s adjusted p-value	Ca_V_3.3-A398V	Dunnett’s adjusted p-value
**window current/ Ipeak**	0.023 ± 0.004	0.0005***	0.023 ± 0.004	>0.9999	0.000 ± 0.000	0.0300*
**window current Vmax**	-47.8 ± 0.819	<0.0001****	-55.4 ± 1.546	0.0001***	--	--
			**Ca**_**V**_ **3.3-M1425V**	**Dunnett’s adjusted p-value**	**Ca**_**V**_ **3.3-M1425I**	**Dunnett’s adjusted p-value**
**window current/ Ipeak**	--	--	0.035 ± 0.007	0.4082	0.044 ± 0.010	0.0490*
**window current Vmax**	--	--	-56.1 ± 1.135	<0.0001****	-54.1 ± 1.092	0.011**

Displayed are means±SEM, Dunnett’s adjusted p-values of one respective Ca_V_3.3 variant vs. WT, and the one-way ANOVA p-values of WT vs. the four Ca_V_3.3 variants. *p < 0.05, **p < 0.01, ***p < 0.001, ****p < 0.0001

### Increased versus decreased neuronal firing in a model of TRN neurons

**Fig 6:**
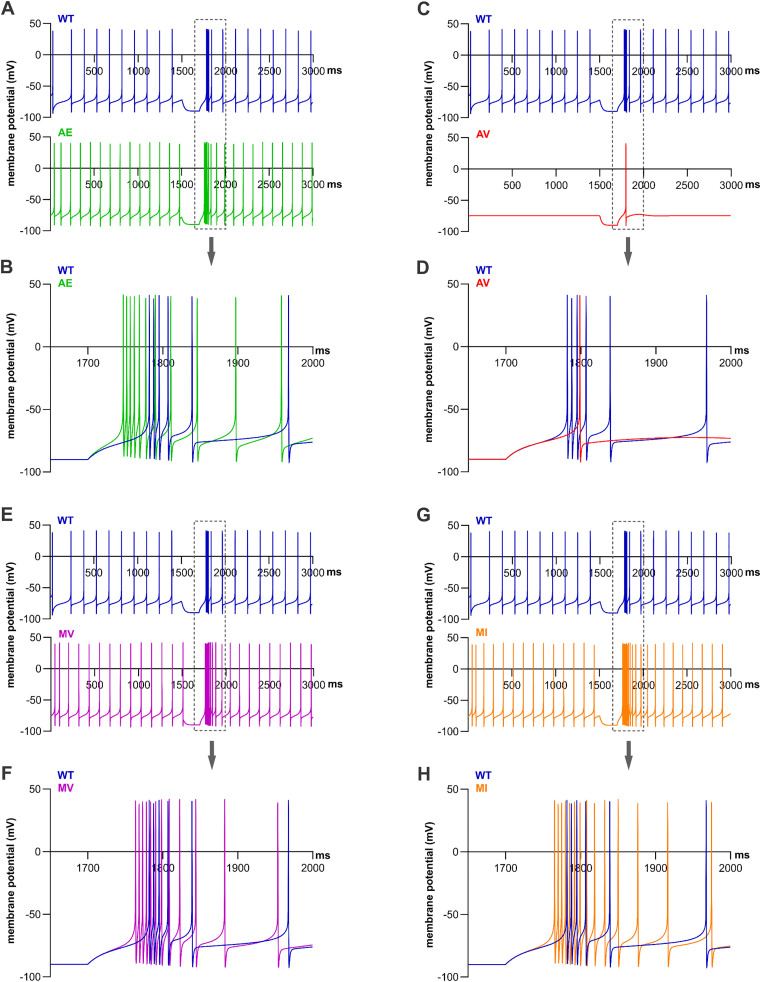
NEURON computer modelling predicts increased electrical activity at rest and in response to hyperpolarizing pulses for three variants of Ca_V_3.3 and quiescence at rest for the A398V variant. (**A**, **C**, **E**, and **G**) Show the spontaneous firing at rest of each variant compared to WT for 1500 ms. At 1500 ms, a 200 ms hyperpolarizing pulse (-0.088 nA) to -90 mV was injected to test rebound firing. (**B**, **D**, **F**, and **H**) Show zoom-ins of 350 ms right after the hyperpolarizing pulse. (**A**, **E**, and **G**) A398E (AE, green), M1425V (MV, magenta), and M1425I (MI, orange) show a two Hz increased firing frequency at rest (9 Hz) as compared to WT (7 Hz). **(C)** A398V (AV, red) does not fire action potentials at rest. (**B**, **D**, **F**, and **H**) Both wildtype and variants show an increased firing rate after the hyperpolarization to -90 mV; AE, MV, and MI fire with a higher frequency than WT. AV only fires a single action potential.

Ca_V_3.3 channels are highly expressed in thalamic reticular nucleus (TRN) neurons. To investigate the effect of Ca_V_3.3 variants on TRN neuron excitability we performed computer modelling in the NEURON [16] platform using the previously published models [17]. We entered the experimentally determined values for the time constants of activation and inactivation, and the V_1/2_ and slope of activation and inactivation in the computer model to generate calcium currents with biophysical properties closely resembling the kinetics and voltage dependence of activation and inactivation as experimentally measured. As all *CACNA1I* disease-associated variants were present in the heterozygous state in the patients, we adapted the model to imitate an equal expression of the two *CACNA1I* alleles. Thus, the current function in the model is composed of two components, one with the properties of wild-type Ca_V_3.3 and one with the properties of the respective variant. Without applying direct current, the model cell containing the Ca_V_3.3-WT has a resting membrane potential of -65 mV and shows spontaneous firing with a frequency of 7 Hz (Fig 6 and Table 5).

**Table 5:** NEURON computer model.

	RMP TRN neuron		hyperpolarizing current inj. to -90 mV		RMP -90 mV
	actual RMP	spontaneous freq.	# rebound peaks	max. rebound freq.	rheobase
**WT**	-65 mV	7 Hz	3-5	169.5 Hz	0.065 nA
**AE**	-75 mV	9 Hz	7-11	222.2 Hz	0.051 nA
**AV**	-75 mV	–	2-4	–	0.073 nA
**MI**	-75 mV	9 Hz	12-16	204.1 Hz	0.050 nA
**MV**	-75 mV	9 Hz	12-16	222.2 Hz	0.051 nA

Interestingly, we observed that the three variants with gain-of-function effects (A398E, M1425V, and M1425I), as well as the variant with loss-of-function effects (A398V), shifted the resting membrane potential from -65 mV to -75 mV (Fig 6 and Table 5). By changing one parameter at the time, the model revealed that this is mostly due to the left-shifted voltage dependence of inactivation.

Secondly, modelling the Ca_V_3.3 variants revealed that the frequency of spontaneous firing differed between wild-type and variants. The spontaneous firing frequency of the three variants with gain-of-function effects (A398E, M1425V, and M1425I) was 9 Hz; representing a 2 Hz or close to 30% increase compared to the WT frequency (Fig 6A, 6E, and 6G, and Table 5). Consistent with the essential role of Ca_V_3.3 currents for TRN rhythmicity, spontaneous firing was completely absent when modelling the loss-of-function variant A398V (Fig 6C). After injecting a positive current to hyperpolarize the membrane for 200 ms at -90 mV, the rebound firing frequency was transiently increased in all models (Fig 6B, 6D, 6F, and 6H). However, firing frequency of the models containing the Ca_V_3.3 gain-of-function variants showed an increased frequency (Table 5). For A398V, the hyperpolarizing pulse initiated only a single action potential after which the cell stopped firing altogether (Fig 6D).

**Fig 7:**
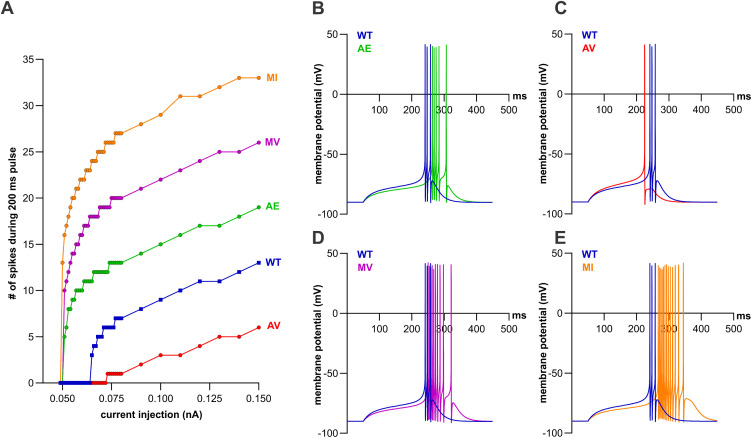
Three variants lower the rheobase and increase firing frequency of Ca_V_3.3, while the A398V variant shows the opposite effect. The resting membrane potential was set to -90 mV and 200 ms depolarizing current pulses of increasing amplitudes were applied. **(A)** A lower current injection necessary to stimulate an action potential (rheobase) was observed for A398E (AE, green, 0.051 nA), M1425V (MV, magenta, 0.051 nA), and M1425I (MI, orange, 0.050 nA) and all three fire with a higher frequency at every pulse, as compared to wild-type (WT, blue, 0.065 nA). In the model, A398V (AV, red, 0.073 nA) has lower firing frequencies and higher rheobase than WT. **(B-E)** Electrical activity at the rheobase. AE, MV, and MI show an increased latency to the start of action potentials compared to WT, while AV shows a faster rising phase and shortened latency than WT.

Another measure of the increased excitability of the TRN neurons is the minimum depolarizing current necessary to trigger firing, i.e., the rheobase. To determine the rheobase, we injected incrementally increasing depolarizing current for 200 ms starting from a resting membrane potential of -90 mV. For A398E and M1425V, firing commenced at current injections of 0.051 nA and for M1425I at 0.050 nA, each substantially lower than the rheobase of 0.065 nA of WT (Fig 7A, 7B, 7D, and 7E, and Table 5). Moreover, at every tested current injection the firing frequencies of these three variants were higher than in the wild-type model (Fig 7A). Contrarily, we found a loss-of-function effect for A398V with an increased rheobase of 0.073 nA and decreased firing frequencies at every tested current injection above threshold (Fig 7A and 7C, and Table 5).

### Structure modelling predicts changes in side-chain interactions and hydrophobicity of the Ca_V_3.3 activation-gate

**Fig 8:**
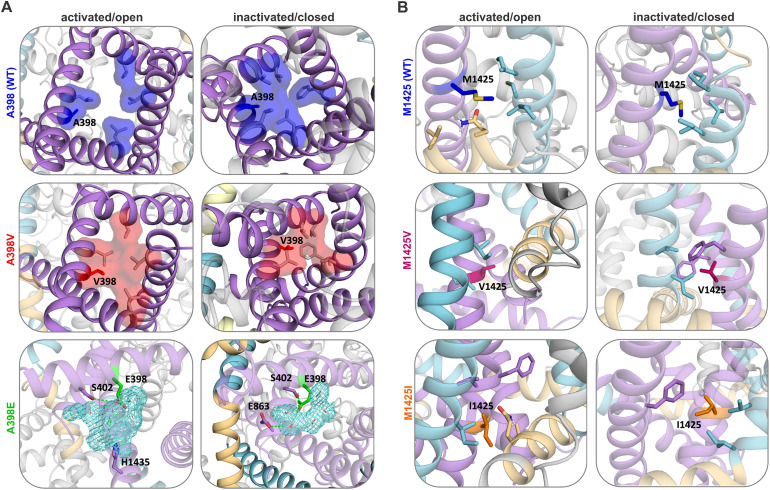
Structure model predictions of wildtype and A398E/V and M1425V/I Ca_V_3.3 channels. Shown is the comparison between the wild-type structure model and the four variants in the open pore and closed pore states. **(A)** A398 points into the gate aperture. The clouds, blue in A398 (WT) and red in A398V, show the hydrophobic surface. The side chain of V398 (red) forms a hydrophobic plug (red cloud) together with other hydrophobic residues in IS6 and neighbouring S6 helices, sealing the gate even in its open configuration. E398 (green) forms hydrogen bonds with residues in IS6 (S402) and neighbouring S6 helices, as well as a water network (blue mesh) inside the gate. **(B)** The two variants of M1425 can both form stabilizing hydrophobic interactions with neighbouring hydrophobic residues pointing away from the gate aperture.

In order to explain the observed changes in biophysical properties of the Ca_V_3.3 variants on a structural level, we performed molecular dynamic simulation of the Ca_V_3.3 α1-subunit bearing the different mutations. In addition to our model of the cryo-EM structure of the Ca_V_3.3 α1-subunit in the inactivated state (VSDs up/gate closed) (PDB accession code: 7WLI) [18], we generated a homology model of Ca_V_3.3 with an open gate (VSDs up/gate open) based on the available Na_V_1.5 α1-subunit open pore structure [19]. Both mutated residues (A398 and M1425) are located at the intracellular end of S6 helices, surrounded mainly by hydrophobic residues (Fig 8A and 8B). Whereas the side chain of A398 points directly into the aperture of the gate, the side chain of M1425 is oriented away from the gate towards the IIIS4-S5 linker and the IIIS5 helix (Fig 1B inset). This indicated two distinct functions of the two residues in wild-type Ca_V_3.3 as well as distinct effects of the variants on structure and function of the gate. The polarity of the side chain at position 398 affects the hydration status of the gate and thus ion permeation directly. In contrast, the propensity of the side chain at position 1425 to interact with neighboring residues is expected to affect the mobility of the gate and thus ion permeation indirectly.

While the side chain of wild-type A398 will not form interactions with neighboring residues, substituting the alanine with a glutamate (E398) allows the formation of long-lasting hydrogen bond interactions with neighboring S6 helices in both the activated and inactivated state (Fig 8A). To quantify the effect of the A398E variant on the gate, we calculated electrostatic interaction energies of A398, E398 and V398 (S2 Table). The electrostatic interaction energy is typically negative when the interaction is attractive, indicating an energetically favorable interaction. Thus, the increase in interactions of E398 with the rest of the protein is reflected in a significantly increased interaction energy and reflects increased stabilization of both the activated (open) and inactivated (closed) state. This could underlie the experimentally observed facilitated activation and inactivation of A398E compared to wild-type Ca_V_3.3 (Figs 2 and 4). Water-mediated interactions support the stabilizing effect of the A398E substitution. The increased hydration of the pore is pronounced in the substantial decrease in interaction energy of residue E398 with water of approximately 80 kcal/mol compared to the WT (S2 Table).

Whereas A398E results in an increase in water and interaction networks, A398V reduces the access of water in the gate and forms a hydrophobic cloud/plug by interacting with the hydrophobic residues in the surrounding S6 helices, thereby partially sealing the pore (Fig 8A). The increase in hydrophobicity in the gate affects ion permeability and could explain the striking current reduction observed experimentally (Fig 2F and 2G).

On the contrary, changing residue M1425, which points away from the pore, to either an isoleucine or valine does not directly affect the hydrophobicity and properties in the pore. Instead, it results in the additional stabilization of both the activated and inactivated states by forming stronger and more stable hydrophobic interactions of S6 with the S4-S5 linker and the S5 helix (Fig 8B). This is expected to affect the mobility of the gate-forming helices. Methionine at this position allows for a higher mobility reflected in a higher B-factor compared to that of the two amino acid changes (S3 Table). Isoleucine has a higher hydrophobicity compared to valine, resulting in a stronger hydrophobic interaction network stabilizing the helices forming the gate.

## Discussion

Here, we report three heterozygous *CACNA1I* missense variants p.(Met1425Val), p.(Ala398Val) and p.(Ala398Glu) as novel disease-causing variants for a neurodevelopmental disorder with or without seizures.

Variants A398E and A398V affect the same amino acid residue in the S6 helix of the first domain of the pore forming α1 subunit of Ca_V_3.3. The third variant, M1425V, affects the same IIIS6 amino acid residue that was substituted in one of our previously described activation-gate modifying *CACNA1I* disease-associated variants, M1425I [9]. In this study, in an attempt to shed light on disease mechanisms, we compared the effects of two different substitutions of A398 and M1425 on channel structure, function, and cellular excitability. Interestingly, while the pair of M1425 substitutions consistently showed comparable effects on the biophysical properties of Ca_V_3.3, the two substitutions of A398 behaved remarkably different and had opposite effects on channel function. Electrophysiological analysis revealed that variants A398E, M1425I, and M1425V result in gain of channel function effects, while A398V consistently showed gating defects leading to a partial loss of channel function. For the gain-of-function variants, the voltage dependence of activation and inactivation was left-shifted. Moreover, we observed increased closing time of the channel gate, apparent by slowing of the inactivation and deactivation kinetics of these variants. In the wake of an action potential, this leads to increased calcium influx. This increased calcium influx can lead to calcium toxicity in the developing nervous system that could contribute to the neurodevelopmental disorder in the patients. Interestingly, as opposed to the other three variants, A398V accelerates both channel inactivation and deactivation. Naturally, the faster channel closing of A398V channels results in a decreased calcium influx upon activation. Additionally, A398V has an almost 3-fold decreased current density compared to Ca_V_3.3 wild-type. This shows for the first time that not only increased but also reduced calcium influx through Ca_V_3.3, a partial loss of channel function, can underlie *CACNA1I*-related ID/DD in patients.

Not only in the wake of an action potential, also during rest there are changes in calcium influx through the mutated Ca_V_3.3 channels investigated here. T-type channels activate and inactivate close to the resting membrane potential of neurons [20]. The incomplete overlap of calcium current activation and inactivation leads to a considerable percentage of the channels being constantly active, thus producing a persistent leak current at rest. This window current through Ca_V_3.3 channels is sufficient to depolarize the membrane to a voltage that triggers low threshold calcium-driven action potentials [21]. Therefore, when this leak window current is increased or shifted closer to the resting membrane potential, this not only makes neurons susceptible for a toxic calcium load, but the window current also directly affects the neuronal excitability. As expected, we observed a left shifted window current for A398E, M1425I, and M1425V. Moreover, for the substitutions of M1425, the leak current was also increased. On the contrary, in Ca_V_3.3-A398V expressing cells the window current was completely abolished, most likely due to the strongly decreased calcium conductance of this variant. Considering the prominent changes in the four studied Ca_V_3.3 channel variants, we expected that both gain and the loss of function variants affect neuronal excitability.

Indeed, when we simulated the effects of the four Ca_V_3.3 channel variants in the NEURON computer model for TRN neurons, we observed opposite effects on excitability. The gain-of-function variants M1425I, M1425V, and A398E all increased the excitability, evident by a lower rheobase and firing with higher frequencies compared to wild-type Ca_V_3.3. The increased firing frequency increases the calcium influx into the cell. Thus, the expected toxic calcium load is a combination of increased firing frequency and increased calcium influx per action potential, due to the slowed closing kinetics of the channel. In contrast, A398V fired at a higher rheobase with a lower frequency and showed a complete absence of spontaneous firing at rest. The hyperexcitability of TRN neurons expressing the gain-of-function variants correlates with the seizure phenotype in the patients with the M1425I and A398E variants. In contrast, the 25-year-old patient with the M1425V variant had not yet developed seizures, but experienced staring spells for most of his life and had behavioral abnormalities and abnormal experiences of reality, such as hallucinations and psychosis. Seizures were also absent in the two unrelated patients with the A398V variant, correlating with the lack of hyperexcitability in the A398V NEURON model. How can we explain different substitutions of the same amino acid residue having such tremendously different effects on channel gating properties?

The channel activation-gate of Ca_V_3.3 is lined with mostly hydrophobic residues that point into the gate aperture and interrupt the aqueous ion conduction pathway in the closed pore conformation; one of these residues is A398 [18]. Thus far, no open pore cryo-EM structures of voltage-gated calcium channels are available. However, recent studies comparing the open and closed pore structures of voltage-gated sodium channels implicate that the opening and closing of voltage-gated ion channels depends on hydrophobic side chains slightly turning away from the gate aperture, allowing hydration and thus ion permeation [19,22]. When we modelled the A398V substitution, we saw that due to the introduction of the bulkier and more hydrophobic valine residue, the hydrophobic surface inside the gate increased and pore hydration decreased. This correlates very well with the strong current reduction that we observed for A398V. On the other hand, substituting A398 with a glutamate residue increases the hydration of the pore in our Ca_V_3.3 structure model and allows for water-meditated interactions between E398 and neighboring S6 residues. These interactions increase the stabilization of both the open and closed pore conformation as compared to the wild-type Ca_V_3.3 structure, which could explain the hyperpolarized activation and inactivation curves that we measured for A398E. Moreover, the newly introduced stabilizing interactions could also make the gate structure of A398E less flexible, possibly explaining the slower inactivation and deactivation kinetics observed for this variant.

The two substitutions of M1425 showed the same gating changes as A398E, yet the structural impact is quite different. The side chain of M1425 does not point into the gate aperture but rather outwards forming hydrophobic interactions with the neighboring S5 helix and S4-S5 linker. Replacing M1425 with isoleucine or valine, which have more hydrophobic and less flexible side chains, increases the strength of these interactions. The stabilizing power of these hydrophobic interactions introduced by I1425 or V1425 can underlie the observed stabilization of both the activated and inactivated state, and the slower gating kinetics.

Introducing stabilizing interactions in both the activated and inactivated state in the gain-of-function variants M1425I, M1425V and A398E correlates with shifts of both the voltage dependence of activation and inactivation to more negative potentials. Whereas the partial loss-of-function variant A398V shows a left shift in the voltage-dependence of inactivation but a right shift in the voltage-dependence of activation. An explanation for this could be that, due to the increased hydrophobicity in the gate, the closed state would be more stabilized and even in the activated state, the hydrophobic plug appears to hampers ion conduction.

The phenotype of the previously reported patient with the gain-of-function variant M1425I was more severe than that of the patient with the M1425V variant reported here. The patient with M1425I showed DD, seizures since the age of 2 years, hypotonia, no speech, delayed motor development, cortical visual impairment, and EEG and brain imaging abnormalities [9]. In contrast, the patient with the M1425V variant had only moderate ID and behavioral abnormalities; expressive and receptive language skills were in the normal range. Interestingly, both patients had staring spells (Table 1) [9]. Of note, the differences in clinical severity between the patients with the M1425I and M1425V variants correlate with the more severe effects of M1425I on channel gating as compared to M1425V. The more stabilizing the interactions are, the more severe the gain of channel effects.

The activation gate, and specifically the cytoplasmic end of the S6 helices, is a hotspot for pathogenic variants in genes encoding voltage-gated channels [10]. The M1425 residue in Ca_V_3.3 is part of the highly conserved MFV sequence in the third domain S6 helix of the T-type channel family and corresponds to the M1531 and M1549 residues in Ca_V_3.1 and Ca_V_3.2, respectively. A **de novo* CACNA1G* variant p.(Met1531Val) was detected in a patient with severe epileptic encephalopathy and global cerebral atrophy [23]. The heterozygous *CACNA1H* variants p.(Met1549Ile) and p.(Met1549Val) were associated with early-onset hypertension and hyperaldosteronism [24,25]. The M1531V variant in *CACNA1G* and the two M1549 variants in *CACNA1H* were characterized as gain-of-function variants with left-shifted voltage dependence and slowed inactivation kinetics [23–25], similar to what we observed for the two M1425 variants in *CACNA1I*. The voltage shifts the M1549I variant are more severe than for M1549V [24,25], in line with what we found for M1425I/V in *CACNA1I*.

The A398E gain-of-function variant, in terms of patient phenotype and channel gating changes, fits in well with the other gain-of-function S6 variants in Ca_V_3.3 described by us and by others in the rest of the T-type channel family. There are no reported disease-associated variants in other voltage-gated calcium channels exactly corresponding to the A398 variant. In *CACNA1E* the residue neighboring the corresponding A398 is mutated. The *de novo* variant p.(Gly352Arg) was reported in nine individuals with seizures, developmental delay, and movement disorders. It was found that this variant slowed the kinetics of inactivation and left shifted the voltage dependence of activation, but right shifted the voltage dependence of inactivation [26,27], corresponding to our observation of opposite shifts of activation and inactivation in the A398V variant. Raybaud et al. suggest that G352R stabilizes the open state of Ca_V_2.3 and therefore left shifts the activation but right shifts the inactivation, in line with our interpretation of a stabilized closed state of Ca_V_3.3 A398V underlying the right-shifted activation in combination with the left-shifted inactivation. Moreover, the *CACNA1A *de novo** variant A713T in the IIS6 helix is in a homologous position to one amino acid removed from A398 in IS6. A713T was found in a patient severe developmental epileptic encephalopathies (DEE) including seizures, severe intellectual disability, hypotonia, and ataxia [28,29]. Similar to the *CACNA1I* A398E variant, *CACNA1A* A713T shows gain-of-function effects on the voltage dependence of activation and the kinetics of inactivation. In the same study two other DEE-linked *CACNA1A de novo* variants were found to result in loss-of-function effects, in line with our findings that both loss and gain of channel function can lead to similar neurodevelopmental defects [28].

Remarkably, A398V characterized here, represents the first report of a partial loss-of-function *CACNA1I* variant linked to DD and ID. In the other members of the T-type channel family only one other loss-of-function *de novo* variant linked to DD was very recently reported for *CACNA1G* [30]. Variant M197R is located in the IS4-S5 linker of Ca_V_3.1 and was found in two patients with motor and speech delay with or without seizures, but no intellectual disability. When tested in HEK cells, M197R resulted in a severe current reduction, faster activation kinetics, slowed recovery of inactivation, and a right-shifted (not significant) voltage dependence of activation and inactivation. Other (partial) loss-of-function variants in genes encoding Ca_V_3.1 and Ca_V_3.2 channels are associated with autism spectrum disorder (ASD), epilepsy, or neuromuscular disease [31–35]. However, none of these variants is located in an S6 helix. In *CACNA1I,* other partial loss-of-function variants, outside the activation-gate, were reported as risk variants for schizophrenia. The heterozygous *de novo* variant p.(Arg1346His) in the IIIS5-S6 linker, reduces the current density of Ca_V_3.3 due to hampered membrane expression [2,6]. In a 2022 study on rare missense variants of *CACNA1I* in a schizophrenia cohort by Baez-Nieto et al., four partial loss-of-function variants were reported. The shifts in voltage dependence in these variants are all below 4 mV, but two variants (C1498R in IVS1 & A1553T in IVS2-S3 linker) show a current reduction of approximately 50% [4].

In our previous study on gain-of-function *CACNA1I* variants linked to neurodevelopmental disorders with and without epilepsy, we suggested two parallel disease mechanisms that could explain the patients’ phenotypes [9]. On one hand, neurons expressing Ca_V_3.3 channels with gain-of-function effects, such as left-shifted voltage dependence of activation and window current, increase the excitability of Ca_V_3.3 expressing neurons. Ca_V_3.3 is highly expressed in TRN neurons where they control rhythmic oscillations underlying the NREM sleep spindles [2,3]. Previously, a link of altered T-type calcium currents in TRN neurons to seizures has been established [36,37]. Here the simulation of action potentials in TRN neurons demonstrates that, even when co-expressed with wild-type in a 1:1 ratio, corresponding to the heterozygous genotype in the patients, the gain-of-function Ca_V_3.3 variants cause hyperexcitability. This data suggests that the Ca_V_3.3 gain-of-function variants that we report in this and in our previous study underlie the seizure phenotype in the patients. On the other hand, all gain-of-function variants experienced increased calcium influx at rest and in the wake of an action potential due to hyperpolarized window currents and slowed channel closing time, respectively. This increases the calcium load of neurons from early development on, and thus can induce calcium toxic defects resulting in altered synapse formation, cell differentiation, and even cell death. Together, these cellular defects could underlie DD/ID in patients with heterozygous *CACNA1I* pathogenic variants.

On the contrary, the partial loss of channel function of A398V leads to decreased calcium load, due to faster channel gate closing time and reduced current density; yet this variant is also associated with DD/ID in two individuals. Thus, both gain and partial loss of Ca_V_3.3 channel function can lead to neurodevelopmental phenotypes. Although not in the T-type channel family, in the broader voltage-gated calcium channel family there are several loss-of-function variants linked to ID/DD (reviewed in [38]). Generally, loss-of-function variants in voltage-gated calcium channels are linked to less severe cases of ID/DD, and epilepsy is less often a comorbidity compared to gain-of-function variants linked to ID/DD. Several cellular mechanisms have been proposed to explain how decreased calcium influx in neurons could underlie ID/DD (reviewed in [38]). One possibility is the autophagy pathway, activated in the cells’ mitochondria in response to low calcium levels leading to metabolic stress due to reduced ATP synthesis [39]. Moreover, Ca_V_3.3 is responsible for healthy NREM sleep spindles and previous research has shown that synaptic plasticity via NMDA receptors in the thalamocortical circuit depends on the rhythmic oscillations controlled by Ca_V_3.3 [2,3,40]. It is therefore well conceivable that distortion of these sleep spindles, due to either increased or reduced Ca_V_3.3 activity, contributes to the neurodevelopmental delay and intellectual disability in the *CACNA1I* patients. Further research in animal models will have to elucidate which pathways, underlying the diverse neurodevelopmental phenotype, are affected by reduced versus increased Ca_V_3.3 activity.

## Materials and methods

### Ethics statement

Genetic studies were performed clinically or as approved by local Institutional Review Boards such as the Ethics Committee of the Hamburg Medical Chamber (PV7038–4438-BO-ff; Hamburg, Germany).

The parents of the patients provided written informed consent for participation in the study, clinical data and specimen collection, genetic analysis, and publication of relevant findings.

### Exome sequencing

#### Patients 1 and 3.

Patient 1: Exome sequencing on DNA samples of patient 1 and his parents was performed using the KAPA HyperExome Probes (Roche) and a HiSeq4000 sequencer (Illumina). Data analysis was performed using RoDa4 pipeline and Alissa v5. In total 76,764,134 unique short reads of 151 bases were generated and uniquely aligned to the human reference genome GRCh37/hg19, generating a mean coverage of 103x per base within the RefSeq protein coding bases of the human genome with >92% of the target covered > 30x. In total 54,066 DNA variants were identified.

Patient 3: Exome sequencing on DNA samples of patient 3 and his parents was performed using the KAPA HyperExome Probes (Roche) and a NovaSeq Xplus sequencer (Illumina). Data analysis was performed using RoDa4 pipeline and Alissa v5. In total 83,307,934 unique short reads of 151 bases were generated and uniquely aligned to the human reference genome GRCh37/hg19, generating a mean coverage of 134x per base within the RefSeq protein coding bases of the human genome with >95% of the target covered > 30x. In total 52,799 DNA variants were identified.

In each patient *de novo* variants (dominant model) and hemi-/homozygous or compound heterozygous variants (recessive model) were analyzed in a trio-based analysis (patient and parents). A large part of the exome (in principle all coding exons and 6 flanking intronic nucleotides) was analyzed. Only clinically significant variants which are associated with the primary clinical concern(s) were reported. There were no other candidate variants in both patients detected. According to the working standard procedures and based on the quality parameters, no validation of the variants using Sanger sequencing was required.

#### Patient 2.

Trio exome sequencing for patient 2 and her parents was carried out as described previously [41]. Briefly, DNA was sequenced on an Illumina system after exome enrichment using the SureSelect Human All Exon V6 kit (Agilent Technologies) by CeGaT. Each captured library was loaded and sequenced on a HiSeq platform (Illumina). Reads were aligned to the human reference assembly (NCBI GRCh38) with the Burrows-Wheeler Aligner. Variants were called using GATK4 (v4.1.9.0) [42] and Strelka2 (v2.9.10) [43] and were annotated using an in-house developed pipeline. Exonic variants and intronic alterations at exon-intron boundaries ranging from -20 to +10, which were absent from public databases or rare (with a minor allele frequency [MAF] <0.1% and no homo- and hemizygotes in public databases) were retained. Trio exome analysis revealed a *de novo CACNA1I* missense variant in patient 2, which was validated by Sanger sequencing. This variant was absent in patient 2’s brother, who had microcephaly (-3.4 z) and developmental delay. In addition, the heterozygous *TUBB2B* (NM_178012.5) missense variant c.1251C > A; p.(Asp471Glu) was detected in patient 2, which was interpreted as a variant of uncertain clinical significance. Segregation of the *TUBB2B* variant by Sanger sequencing identified the variant in patient 2’s mother, who had microcephaly (-2.8 z) but no developmental delay, and in her brother.

### Targeted gene sequencing intellectual disability, epilepsy, and autism panel

#### Patient 4.

Genomic DNA was extracted from patient 4 specimen. The DNA was analyzed by the Intellectual Disability, Epilepsy and Autism (IDEA) gene panel, which includes hundreds of genes implicated in these phenotypes. The coding regions of targeted genes plus ~10 bases of non-coding DNA flanking each exon were covered by capturing these regions using SureSelect Clinical Research Exome hybridization probes (Agilent Technologies). Captured DNA was sequenced on the NovaSeq 6000 using 2x150 bp paired-end reads (Illumina). Quality control metrics were the following: > 97% of target bases were covered at>20x and mean coverage of target bases > 120x. Data analysis and interpretation was performed by the internally developed Infinity pipeline. Variant calls were made by the GATK Haplotype caller [42] and annotated using in house software and Jannovar [44]. Common benign, likely benign, and low quality variants were filtered from analysis. Segregation of the *CACNA1I* variant revealed that the variant was absent in patient 4’s mother.

### Expression plasmids and transfections

The cloning strategy for GFP-Ca_V_3.3 (Genebank ID AF393329) and GFP-Ca_V_3.3-M1425I were previously described [9].

#### GFP-Ca_V_3.3-A398E/V.

To generate the desired constructs, two different mutations of A398 were introduced into GFP-Ca_V_3.3 by splicing by overlap extension (SOE) PCR. First, the cDNA sequence of human Ca_V_3.3 (nt 527–2723) was amplified in separate PCR reactions using GFP-Ca_V_3.3 as the template, employing overlapping primers that introduced the mutations. The two resulting PCR products were then used as templates for a PCR reaction with flanking primers to connect the nucleotide sequences. The final fragment was digested with BamHI and AvrII and ligated into the corresponding sites of GFP-Ca_V_3.3. The flanking primers used for both construct were: BamHI-F: 5´- CCATCCGCACCGTGCGCGTCCTG- 3´ and AvrII-R: 5´- ATGGGGTCCGGCTGCAGT- 3´. The mutagenesis primers were for GFP-Ca_V_3.3-A398E, A398E-R: 5´- GAACTGGGTC**T**CTATGACAACGAGGCACAGGTTGA- 3´ and A398E-F: 5´- CTCGTTGTCATAG**A**GACCCAGTTCTCGGAGACCAA- 3´, while for GFP-Ca_V_3.3-A398V, A398V-R: 5´- GAACTGGGTC**A**CTATGACAACGAGGCACAGGTTGA- 3´ and A398V-F: 5´- CTCGTTGTCATAG**T**GACCCAGTTCTCGGAGACCAA- 3´. The bases that introduced the mutation are highlighted in bold.

#### GFP-Ca_V_3.3-M1425V.

The M1425V mutation was introduced into GFP-Ca_V_3.3 by splicing by overlap extension (SOE) PCR. First, the cDNA sequence of human Ca_V_3.3 (nt 2707–4824) was amplified in separate PCR reactions using GFP-Ca_V_3.3 as the template, employing overlapping primers that introduced the mutations. The two resulting PCR products were then used as templates for a PCR reaction with flanking primers to connect the nucleotide sequences. The final fragment was digested with AvrII and HindIII and ligated into the corresponding sites of GFP-Ca_V_3.3. The flanking primers were AvrII-F: 5´- TCCAGGAAGGCCTGGACA- 3´ and HindIII-R: 5´- CTTCCCAAAGAGCTCCAC- 3´. The mutagenesis primers were M1425V -R: 5´- CACAAACA**C**GTTTAAAACAAAGAAGCTGACGATGAGCAG- 3´ and M1425V-F: 5´- TCTTTGTTTTAAAC**G**TGTTTGTGGGTGTCGTGGTGGAGAAC- 3´. The bases that introduced the mutation are highlighted in bold.

Sanger sequencing (Eurofins Genomics) verified the integrity of the newly generated plasmid.

### Cell culture and transfections

HEK293T cells were cultured in Dulbecco´s modified Eagle medium (DMEM, Thermo Fisher Scientific) supplemented with 10% (v/v) fetal bovine serum (FBS; Merck) and penicillin- streptomycin (100 U/ml and 100 μg/ml, respectively; Thermo Fisher Scientific) and incubated at 37°C in a humidified atmosphere with 5% CO2. HEK293T cells were transfected with 1 μg of plasmid DNA on the day of plating, using FuGENE-HD transfection reagent (Promega) and cultured in DMEM overnight. The cells were used for experiments on the second day after transfection.

### Electrophysiology and data analysis

In the HEK293T cells calcium currents were recorded with the whole-cell patch-clamp technique in voltage-clamp mode using an Axopatch 200A amplifier (Axon Instruments). Patch pipettes (borosilicate glass; Science Products) had resistances between 1.9 and 4.8 MΩ when filled with (mM) 135 CsCl, 1 MgCl2, 10 HEPES, and 10 EGTA (pH 7.4 with CsOH). The extracellular bath solution contained (mM) 2 CaCl2, 165 choline-chloride, 10 HEPES, and 1 Mg-Cl2 (pH 7.4 with CsOH). Data acquisition and command potentials were controlled by pCLAMP software (Clampex version 10.2; Axon Instruments); analysis was performed using Clampfit 10.7 (Axon Instruments) and SigmaPlot 12.0 (SPSS Science) software. The current- voltage dependence was fitted according to I=Gmax*(V−Vrev)/(1+exp(−(V−V1/2)/k) where Gmax is the maximum conductance of the L-type calcium currents, Vrev is the extrapolated reversal potential of the calcium current, V_1/2_ is the potential for half maximal conductance, and k is the slope. The conductance was calculated using G = (− I * 1000)/(Vrev − V), and its voltage dependence was fitted according to a Boltzmann distribution: G=Gmax/(1+exp(−(V−V1/2)/k). Steady-state inactivation curves were fitted using a modified Boltzmann equation: G=(1 – Gmax)/(1+exp((V – V1/2)/k))+Gmax  where V1/2 is the half-maximal inactivation voltage and k is the inactivation slope factor.

### NEURON computer model

We performed the computer modelling in the NEURON simulation environment [16] using the model for thalamic reticular neurons [17] from the model database at Yale University (https://modeldb.science). The electrophysiological properties of the Ca_V_3.3 channels were modelled using Hodgkin-Huxley equations as described previously [17,45]. We used the single-compartment model and adapted the model to mimic heterozygous expression of the variants. The calcium current (ica) is described as followed:


ica = 0.5*ica1+0.5*ica2 

ica1 = gcabar * m1*m1*h1 * (v−carev)

ica2 = gcabar * m2*m2*h2 * (v−carev) 

$$\begin{array}{c} \text{m}{\text{1}}^{\prime }\;=\;(\text{m}\_\text{inf1 }-\text{ m1})\;/\text{ tau}\_\text{m1 } \end{array}$$

$$\begin{array}{c} \text{h}{\text{1}}^{\prime }\;=\;(\text{h}\_\text{inf1 }-\text{ h1})\;/\text{ tau}\_\text{h1 } \end{array}$$

$$\begin{array}{c} \text{m}{\text{2}}^{\prime }\;=\;(\text{m}\_\text{inf2 }-\text{ m2})\;/\text{ tau}\_\text{m2 } \end{array}$$

$$\begin{array}{c} \text{h}{\text{2}}^{\prime }\;=\;(\text{h}\_\text{inf2 }-\text{ h2})\;/\text{ tau}\_\text{h2 } \end{array}$$

The values of native T-type channels were substituted by the experimentally obtained values for the WT, A398E, A398V, M1425V and M1425I variants. In the case of WT Ca_V_3.3, we used the WT values for both ica1 and ica2.

The equations to model the WT Ca_V_3.3 channel were:


m_inf = 1/(1+exp(−(v+47)/8)

h_inf = 1/(1+exp((v+75)/5.0) 

tau_m = (9+1/(exp((v+25)/10)+exp(−(v+100)/15)))/phi_m 

tau_h = (95+1.0/(exp((v+46)/4)+exp(−(v+405)/50)))/phi_h  

In the case of the variants, we used the WT values for ica1 and the variant values for ica2.

The equations to model the Ca_V_3.3-A398E channel were:


m_inf = 1/(1+exp(−(v+54)/7)) 

h_inf = 1/(1+exp((v+81)/4)) 

tau_m = (6+1.0/(exp((v+25)/10)+exp(−(v+100)/15)))/phi_m 

tau_h = (180+1.0/(exp((v+46)/4)+exp(−(v+405)/50)))/phi_h 

In order to insert the 3-fold current reduction of Ca_V_3.3-A398V specifically, we adapted ica2 for Ca_V_3.3-A398 as follows: ica2 = 0.33* gcabar * m2*m2*h2 * (v−carev).

The equations to model the Ca_V_3.3-A398V channel were:


m_inf = 1/(1+exp(−(v+44)/10.5)) 

h_inf = 1/(1+exp((v+84)/4)) 

tau_m = (4.5+1/(exp((v+25)/10)+exp(−(v+100)/15)))/phi_m

tau_h = (36+1/(exp((v+46)/4)+exp(−(v+405)/50)))/phi_h 

The equations to model the Ca_V_3.3-M1425V channel were:


m_inf = 1/(1+exp(−(v+54)/7)) 

h_inf=1/(1+exp((v+81)/4)) 

tau_m = (11+1.0/(exp((v+25)/10)+exp(−(v+100)/15)))/phi_m 

tau_h=(300+1.0/(exp((v+46)/4)+exp(−(v+405)/50)))/phi_h

The equations to model the CaV3.3-M1425I channel were:


m_inf = 1/(1+exp(−(v+54)/7)) 

h_inf = 1/(1+exp((v+81)/4)) 

tau_m = (11+1.0/(exp((v+25)/10)+exp(−(v+100)/15)))/phi_m 

tau_h = (550+1.0/(exp((v+46)/4) + exp(−(v+405)/50)))/phi_h 

### Molecular dynamics simulations

Structures of the WT Cav3.3 α1-subunit and the variants (A398E, A398V, M1425I, M1425V) were modelled in two different gating states, activated-open (voltage-sensors “up,” activation gate open) and inactivated (voltage-sensors “up,” activation gate closed), by using the available high resolution cryo-electron microscopy (EM) structures of voltage-gated Ca^2+^- and Na^+^-channel pore-forming subunits. The following structures were used as templates: activated-open state: open-stabilized Nav1.5 α-subunit structure (PDB accession code: 7FBS) [19]; inactivated state: Cav3.3 α1-subunit structure (PDB accession code: 7WLI) [18].

Homology modelling has been performed using Rosetta and MOE (Molecular Operating Environment, version 2020.09, Molecular Computing Group Inc., Montreal, Canada). Additionally, ab initio Rosetta was used to generate structures for loops that were not resolved in the original templates [46]. The structures for the variants were derived from the respective wild-type model by replacing the mutated residue followed by a local energy minimization using MOE. The C-terminal and N-terminal parts of each domain were capped with acetylamide and N-methylamide to avoid perturbations by free charged functional groups. The structure was aligned in the membrane using the PPM server [47] and inserted into a plasma membrane consisting of POPC (1-palmitoyl2-oleoyl-sn-glycero-3-phosphocholine) and cholesterol in a 3:1 ratio, using the CHARMM-GUI Membrane Builder [48]. Water molecules and 0.15 M CaCl2 were included in the simulation box. For Ca2 + the standard parameters for Ca2 + -ions were replaced with the multi-site Ca2+ of Zhang et al. [49]; AMPA receptors of [50]; the E protein of SARS-CoV-2 [51], and TRPV channels [52]. All simulations of the WT and mutants were performed using GROMACS 2020.2 [53,54] with the CHARMM36m force field for the protein, lipids and ions (except for Ca2+ as described above) [55]. The TIP3P water model was used to model solvent molecules [56]. The system was minimized and equilibrated using the suggested equilibration input scripts from CHARMM-GUI [57], i.e., the system was equilibrated using the NPT ensemble for a total time of 2 ns with force constraints on the system components being gradually released over six equilibration steps. The systems were further equilibrated by performing a 10 ns simulation no electric field applied. The temperature was maintained at T = 310 K using the Nosé-Hoover thermostat [58], and the pressure was maintained semi-isotropically at 1 bar using the Parrinello-Rahman barostat [59]. Periodic boundary conditions were used throughout the simulations. Long-range electrostatic interactions were modelled using the particle-mesh Ewald method [60] with a cut-off of 12 Å. The LINCS algorithm [61] was used to constrain bond lengths involving bonds with hydrogen atoms. Then molecular dynamics simulations were performed for 1000 ns, with time steps of 2 fs, at 300 K and in anisotropic pressure scaling conditions. Van der Waals and short-range electrostatic interactions were cut off at 10 Å, whereas long-range electrostatics were calculated by the Particle Mesh Ewald (PME) method. PyMOL was used to visualize the key interactions and point out differences in the wild-type and mutant structures (The PyMOL Molecular Graphics System, Version 2.0 Schrödinger, LLC). The interaction energies were calculated using the LIE tool implemented in cpptraj [62].

## Supporting information

S1 Fig WT data of set A398E/V and set M1425V/I pooled for better comparison. (**A**) The current-voltage relationship of the two A398 variants and the two M1425 variants, compared to pooled WT controls. (**B**) Peak current densities are not significantly altered, but reduced by more than 2-fold in AV. For proper statistical values it is necessary to use matched WT controls (same recording day), see Fig 2 and Table 2 in main text. (**C**) Fractional activation curves and (**D**) V_1/2_ of activation scatterplots show significantly left-shifted voltage dependence of activation for Ca_V_3.3 AE by 10.8 mV, MV by 12.0 mV, and MI by 10.7 mV. The V_1/2_ of AV is right-shifted by 3.9 mV, all as compared to pooled wild-type controls. (**E**) Time constants of activation calculated from fits of the rising phase during 500 ms step depolarisations to the indicated voltages. (**F**) Time constants of inactivation determined by fitting the decay phase of currents during 5 s depolarisations. AE, MI, and MV inactivate slower and AV faster, compared to pooled WT. (**G**) Time constants of deactivation determined from tail current decay at indicated repolarising voltages after 15 ms pulse to V_max_. Insets display representative normalized example traces. AE, MI and MV deactivates increasingly slower, A398V faster at -60 mV. Mean ± SEM; p-values calculated with one-way ANOVA and Dunnett’s multiple comparisons test (**A-D**) or with repeated measures ANOVA and the Holm-Sidak test for multiple comparisons (**E-G**); ** p < 0.01, *** p < 0.001, **** p < 0.0001.
(S1_Fig.TIF)

S1 Table Gating properties of *CACNA1I* variants with pooled WT data. Displayed are means±SEM, Dunnett’s adjusted p-values of one respective Ca_V_3.3 variant vs. WT, and the one-way ANOVA p-values of WT vs. the four *CACNA1I* variants. * p < 0.05, ** p < 0.01, **** p < 0.0001.
(S1_Table.XLSX)

S2 Table Interaction energies of the Ca_V_3.3 A398 amino acid substitutions. (S2_Table.XLSX)

S3 Table B-factor values of Ca_V_3.3 M1425 substitutions. (S3_Table.XLSX)
