# Supplementary figures and images for: Two pairs of CACNA1I (CaV3.3) variants with opposite effects on channel function cause neurodevelopmental disorders of varying severity
Source: PLoS Genet. 2025 Aug 18;21(8):e1011828. doi: 10.1371/journal.pgen.1011828 (PMC12396757; doi:10.1371/journal.pgen.1011828)

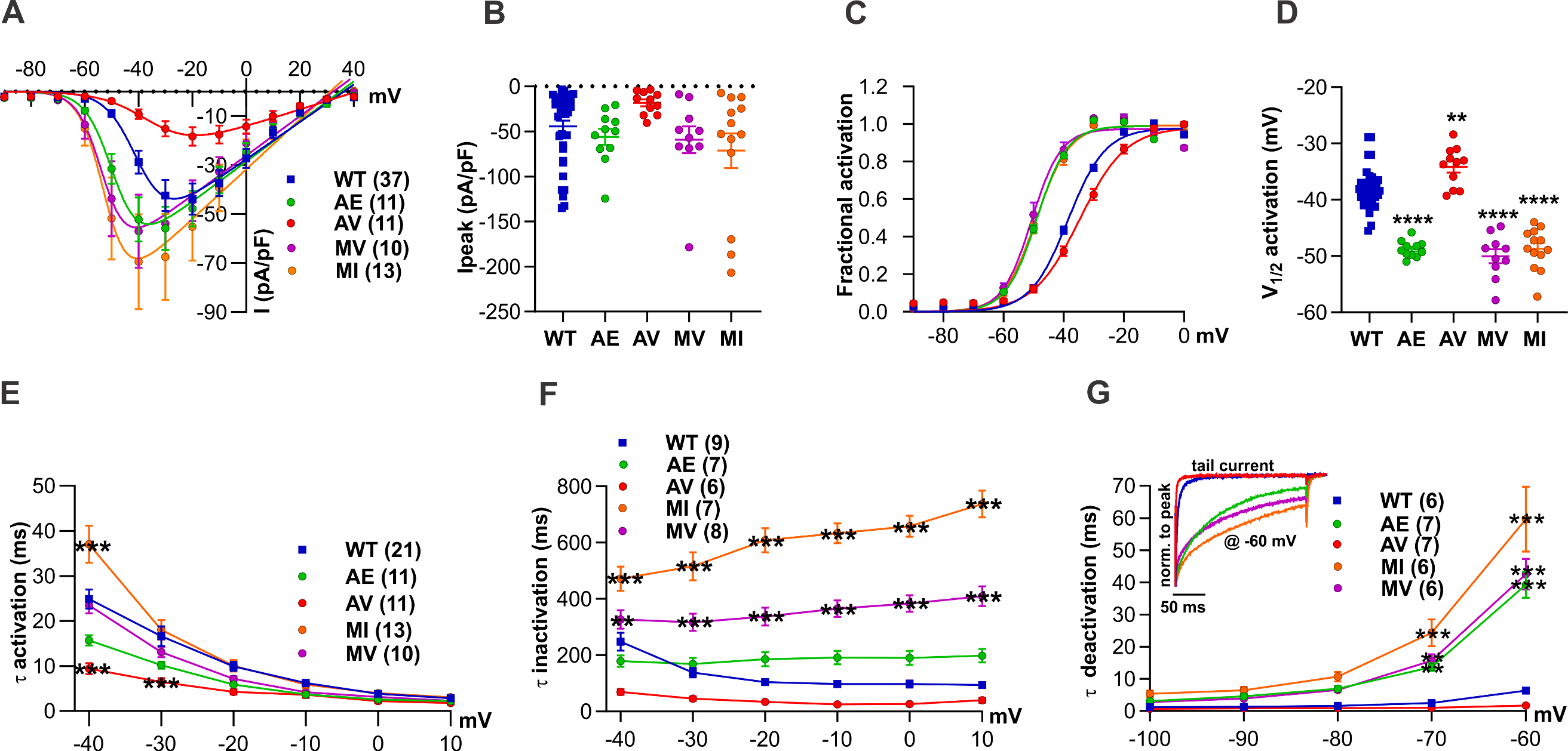

Supplement: S1 Fig — (A) The current-voltage relationship of the two A398 variants and the two M1425 variants, compared to pooled WT controls. (B) Peak current densities are not significantly altered, but reduced by more than 2-fold in AV. For proper statistical values it is necessary to use matched WT controls (same recording day), see Fig 2 and Table 2 in main text. (C) Fractional activation curves and (D) V1/2 of activation scatterplots show significantly left-shifted voltage dependence of activation for CaV3.3 AE by 10.8 mV, MV by 12.0 mV, and MI by 10.7 mV. The V1/2 of AV is right-shifted by 3.9 mV, all as compared to pooled wild-type controls. (E) Time constants of activation calculated from fits of the rising phase during 500 ms step depolarisations to the indicated voltages. (F) Time constants of inactivation determined by fitting the decay phase of currents during 5 s depolarisations. AE, MI, and MV inactivate slower and AV faster, compared to pooled WT. (G) Time constants of deactivation determined from tail current decay at indicated repolarising voltages after 15 ms pulse to Vmax. Insets display representative normalized example traces. AE, MI and MV deactivates increasingly slower, A398V faster at -60 mV. Mean ± SEM; p-values calculated with one-way ANOVA and Dunnett’s multiple comparisons test (A-D) or with repeated measures ANOVA and the Holm-Sidak test for multiple comparisons (E-G); ** p < 0.01, *** p < 0.001, **** p < 0.0001. (S1_Fig.TIF) [file pgen.1011828.s001.tif]
